# Assessed and perceived oral health of older people who visit the dental practice, an exploratory cross-sectional study

**DOI:** 10.1371/journal.pone.0257561

**Published:** 2021-09-24

**Authors:** Pieternella C. Bots-VantSpijker, Claar D. van der Maarel-Wierink, Jos M. G. A. Schols, Josef J. M. Bruers

**Affiliations:** 1 Flemish-Netherlands Geriatric Oral Research Group (BENECOMO), Dutch Association for Gerodontology, Bunnik, The Netherlands; 2 Department of Oral Public Health (OPH), Academic Centre for Dentistry Amsterdam (ACTA), University of Amsterdam and Vrije Universiteit, Amsterdam, The Netherlands; 3 Department of Oral Medicine, Academic Centre for Dentistry Amsterdam (ACTA), University of Amsterdam and Vrije Universiteit, Amsterdam, The Netherlands; 4 Caphri/Department of Family Medicine and Department of Health Services Research, Maastricht University, Maastricht, The Netherlands; 5 Royal Dutch Dental Association (KNMT), Utrecht, The Netherlands; University of the Pacific, UNITED STATES

## Abstract

**Objectives:**

To assess the oral health of older people who visit the community dental practice from both the dentists’ and the patients’ perspective.

**Materials and methods:**

In this exploratory cross-sectional study the oral health of Dutch community dwelling older people was assessed. A representative sample of general dental practitioners was asked to randomly and prospectively select one older patient and describe this patient using a specially-developed registration form; in addition the patient was requested to complete a questionnaire. The oral health of older people was described from the perspective of the dentists and the perspective of the older people themselves based on the definition of oral health from the World Dental Federation (FDI].

Relations between oral health of older people and dentist and older patient characteristics were analysed using Spearman’s rank correlation coefficient (rho) and an ordinal regression model.

**Results:**

In total, 923 dentists were asked to participate in the study; data was available for 39.4% dentist-patient pairs. Dentists assessed the oral health of older patients as good or acceptable in 51.4% of the cases while this was the case in 76.2% of older patients themselves. The assessment of the dentist gets more negative with high treatment intensity and with older patients having certain diseases and more medication, while the assessment is more positive for older patients who visit the dentist on a regular basis.

Older people’s assessment of their oral health gets more negative by being female and with high treatment intensity, having certain diseases and higher use of medication.

**Conclusions and clinical relevance:**

Chronically illness as expressed by the number of diseases and the use of medication, seems to be a risk factor for poor oral health.

Older patients themselves assess their oral health differently, mostly more positive, than their dentist.

## Introduction

In 2016, the FDI World Dental Federation defined oral health as a multifaceted human condition, which offers the ability to speak, laugh, smell, taste, chew, swallow, touch and convey a range of emotions through facial expressions, all with confidence and without pain, discomfort or disease of the craniofacial complex. Oral health is considered to be a fundamental component of physical and mental well-being that is influenced by the values and attitudes of individuals and communities. Furthermore, oral health reflects physiological, social and psychological characteristics that are essential for the quality of life. Oral health is influenced by the individual’s experiences, perceptions, expectations and ability to adapt to the circumstances [1].

This definition gives a broad and holistic description of oral health, whereas oral health assessments are often based on only one or a few items such as caries and periodontitis. Moreover, the assessment from the dentist’s point of view may be different from the patient’s perspective. The dentist looks at caries activity, the condition of the gingiva, mucous membrane and oral function, whereas the patient focuses more on a pain-free, functional and aesthetically acceptable mouth. Several oral health measures have been developed based on the professional approach: The dentist can assess oral health in terms of caries by using the DMFT index (sum of decayed, missing and filled teeth), for periodontal situation the CPITN index (Community Periodontal Index of Treatment Needs) or–in the Netherlands–the DPSI index (Dutch Periodontal Screening Index), or using a combination of the measurements for caries and periodontal situation [2–5]. In addition, there are separate measures for assessing endodontic health and erosion [6–8].

Determining oral health from the patient’s point of view often involves an assessment of the quality of life, with oral health being considered to be an aspect of general health. The Oral Health Impact Profile 7, for example, measures quality of life as related to oral health; the Geriatric Oral Health Assessment Index (GOHAI) is based on this profile, focusing on older patients [9,10]. The patient can also score the degree of pain in or near the mouth by for example using a Numerical Rating Scale (NRS) and Visual Analogue Score (VAS) [11].

Few studies describe oral health from the perspective of both the dentist and the patient. Moreover, most studies describe only some aspects of oral health, usually based on the DMFT index. For instance, the number of dental teeth reported by patients corresponds to the clinical situation observed by the dentist [12,13], but in terms of periodontal oral health the patient’s assessment differs from that of the dentist [14,15]. It is also known in medicine that the patient and the treating physician often assess the health status differently [16,17].

Given these knowledge gaps, the objective of this study is to investigate the oral health of older people living at home from the professional perspective of the dentist as well as from the perspective of the older person. Furthermore, to what extent does the dentist’s assessment match the assessment by older people themselves, and what characteristics of older people are related to each of these assessments?

## Materials and methods

### Study design

This exploratory, cross-sectional study investigates the oral health of community dwelling older people living in their home environment. In the study asked a representative sample of dentists was asked to select one older patient randomly from their files and describe this patient using a specially-developed recording form. In addition the patient was asked to complete a short questionnaire. The design of this study has been described previously [18].

### Recruitment

**Fig 1:**
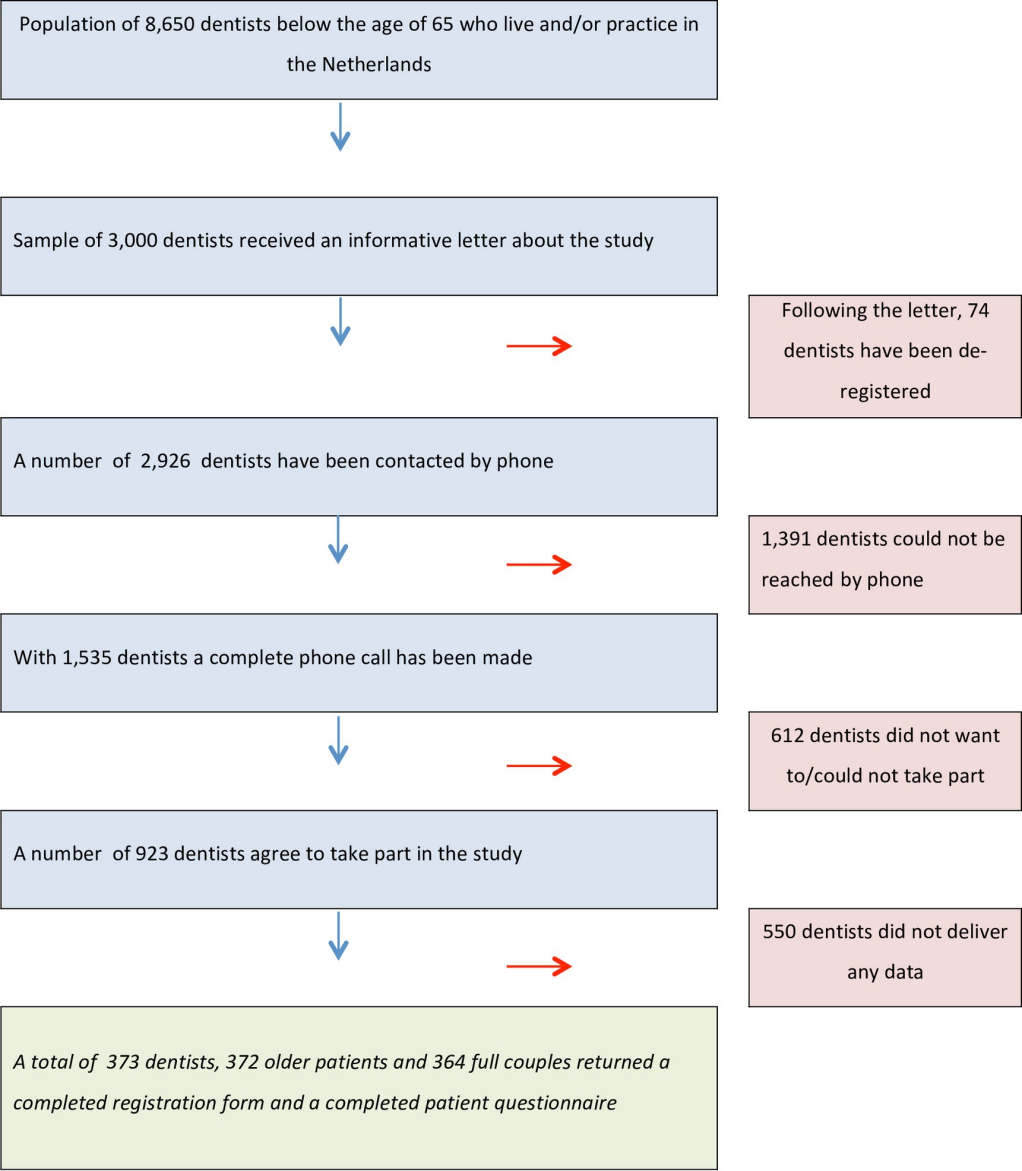
Flowchart of recruitment.

The Royal Dutch Dental Association (KNMT) manages a reliable database of all qualified dentists in the Netherlands. From this database, a random sample of 3,000 dentists was drawn from the 8,656 dentists aged 64 or younger who live and/or work in the Netherlands. These dentists received an information letter about the study stating that they would be contacted by phone within one week for a further explanation of the study. In the end, full phone conversations were held with 1,535 dentists, of whom 923 were willing to participate in the study. Of these, 325 of them were asked to include a patient aged 60 to 64 in the study and 598 were asked to involve a patient aged 75 or older. See Fig 1 Flowchart of recruitment.

The intention was to make a clear comparison between ‘younger’ older people and ‘older’ older people. A patient could be included if he or she had visited the dentist for a check-up or treatment in the past two years. Both the dentist and the patient received an information letter and a registration form (dentist) or a questionnaire (patient) plus an informed consent form.

### Data collection

In addition to the medical history, dental history and data about dental visits in the past, the registration form for the dentists about the older patient in question requested data about morbidity, oral health diagnoses, treatment strategy and treatments used. Additionally, the dentist was asked to give a few characteristics of their practice and the make-up of the dental team.

The patient questionnaire included general data collected from the older patients themselves, such as age, sex, marital status and socioeconomic status (SES), and whether they had supplementary dental insurance. Additionally, data was collected about tobacco use and alcohol consumption, medication, frailty, oral health self-care and dental visits, and about their perceived oral condition and any wishes regarding oral health. A translated recording form for the dentist as well as the patient questionnaire can be requested from the first author.

### Data construction oral health

To attain a measure for the oral health of older people who still visit dental practices, the definition of oral health from the FDI was used [1]. This definition has three main themes: disease, pain and discomfort. Different data related to aspects of these themes was selected from both the dentist registration form and the patient questionnaire. If necessary, each data item was recoded to a dichotomous variable, that is whether a particular aspect was present or absent.

**Fig 2:**
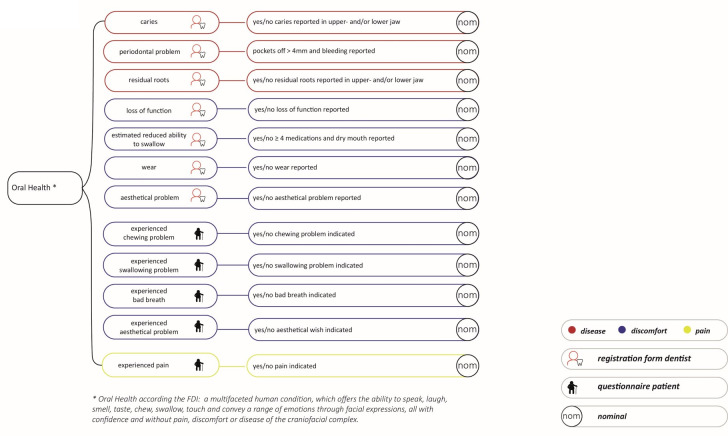
Aspects oral health.

The main theme ‘disease’ included the aspects caries activity, periodontal disease and residual roots. The main theme ‘pain’ included the aspect of pain in or near the mouth. The main theme ‘discomfort’, looked at the general function, chewing and swallowing, bad breath and aesthetics. Fig 2 summarises the various aspects for each main theme, also stating whether the data was obtained via the dentist registration form or the patient questionnaire. The ability to chew, determined by the dentist and based on occlusal units, was disregarded because over a third of the 371 dentists had no indicative or reliable data.

It was assumed that not all seven aspects would be equally important to the dentist for determining the oral health of an older patient, and by analogy not all of the five aspects would be equally important to older patients for their perceived oral health. Therefore the items were weighted before counting. For the ‘dentist aspects’, this weighting was based on average ranking numbers, obtained by asking 23 random dentists (non-participants) to rank the seven aspects in order of importance for an older patient’s oral health. Likewise, ten older persons (non-participants) were asked to do the same for the five ‘patient aspects’ [19]. Appendix S1 Table gives the average rank for each aspect as well as the derived weight. By adding up the weighted scores, an overall score for the oral health of an older patient as assessed by a dentist and an overall score for the oral health as perceived by the patients themselves, were obtained.

Spearman’s rank correlation coefficient (r_s_) was used to assess reliability by comparing a dentist’s sum score with the single score that a dentist was asked to give about the overall oral health of the patient. Similarly, the sum score obtained from a patient was compared with the single score that older patients were asked to give for their own oral health.

### Data construction patient characteristics

**Fig 3:**
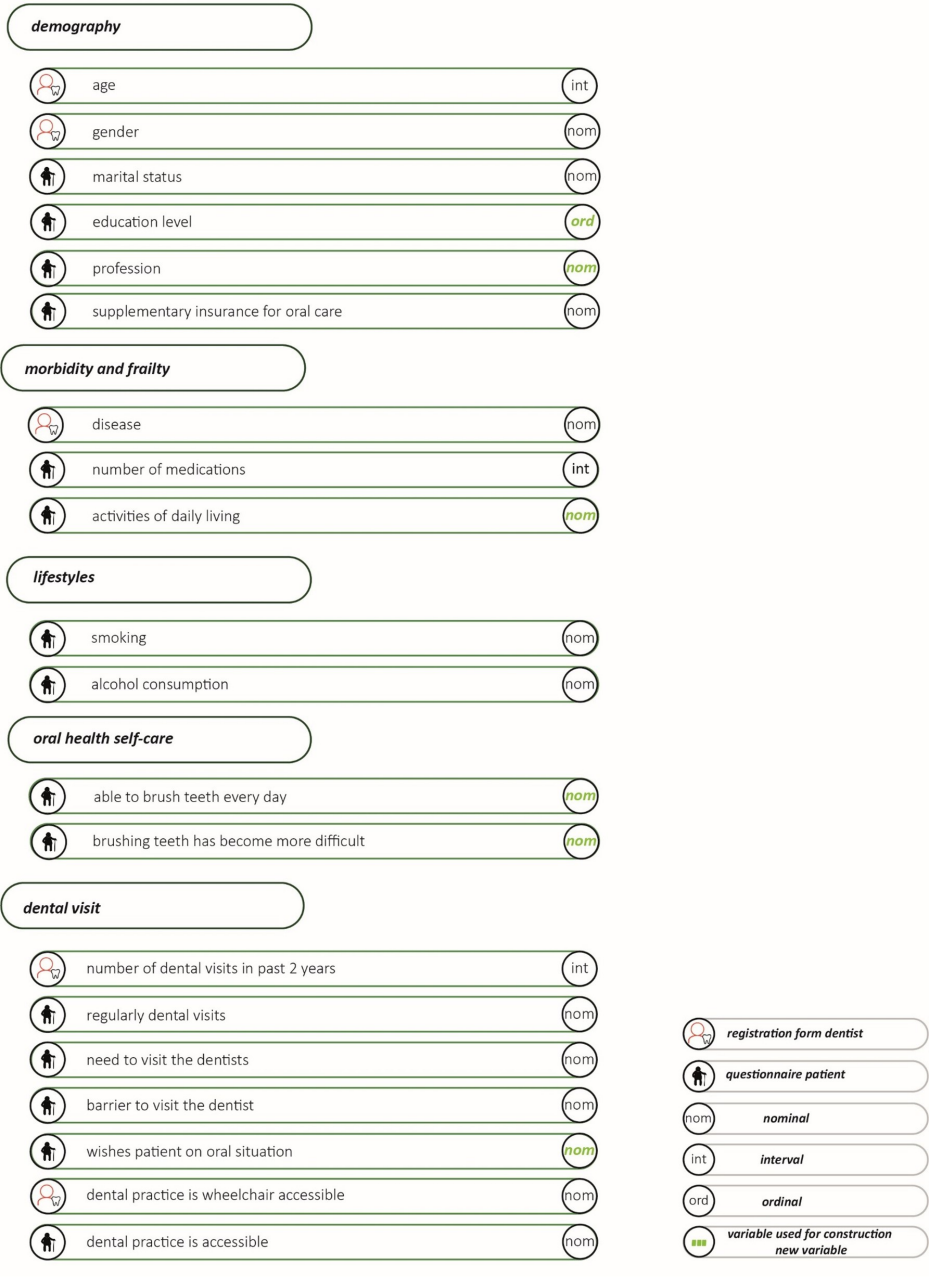
Overview of variables used in analysis.

Fig 3 summarises all the variables used in the analysis, including the level of measurement (nominal, ordinal or interval). It also states whether the variable was obtained via the dentist registration form or the patient questionnaire.

- Using data from the patient questionnaire, the SES of older people was determined based on their highest level of education (low/average/high) and/or their profession using the ISCO classification [20].
- Frailty is often defined on the basis of clinical tests [21–25]. For the sake of feasibility in this study—which is based on data from questionnaires, based on routinely available data and self-administrated data—we chose a simple classification of the ability to carry out ‘Activities of Daily Living’ (ADLs) [26], derived from the known measures of frailty. This was determined as a sum score of seven dichotomous items about mobility, care dependency and care support (Cronbach’s alpha = 0.756). An older person was deemed frail if he or she affirmatively was responded to three or more questions.
- Capability for oral health self-care was determined on the basis of whether or not the older person was able to brush their teeth every day and whether or not brushing had become more difficult.
- The various wishes of older patients associated with their oral status were recoded into whether or not there was a wish to improve their current oral status.

### Statistical processing

All data was processed, linked and analysed using the statistical software package SPSS (IBM Corp, 2016). First, the distribution of the oral health measures as constructed was determined (procedure ‘FREQUENCIES’). This was followed by a bivariate analysis to determine which characteristics of older patients (in terms of demographics, morbidity and frailty, living habits, oral care behaviour and dental visits) are related to the oral health measures given by both the dentist and the older patient. This was done using Spearman’s rank correlation coefficient (rho) (procedure ‘CORRELATE’).

After that, an analysis was performed using an ordinal regression model to determine which characteristics of older people ultimately determine their oral health as assessed by the dentist and the older patient. To that end, the initial models included the characteristics that had a significant bivariate correlation (p < 0.10) with that assessment. The final models were produced using the remaining characteristics after stepwise elimination of non-significant characteristics. Both final models offered a significantly better estimate than the baseline intercept-only models. The ‘goodness of fit test’ for both models showed no significant deviation from the null hypothesis and the good adjustment measure (procedure ORDINAL REGRESSION).

### Ethical approval

In the Netherlands, according to the Medical Scientific Research involving Human Subjects Act (WMO) for research in which research data are obtained by studying medical records, a non-WMO declaration is desirable. The Medical Ethical Committee (METc) of the Free University Amsterdam (Vrije Universiteit Amsterdam) has provided such a non-WMO-declaration in July 2016 (016.193: Bots-van ‘t Spijker Title: ‘De zorg aan oudere patiënten in de tandartspraktijk’). To guarantee the confidentiality for the processing of the data for this study and to safeguard the anonymity of the participants, an independent research agency (’Third Party Research Institute) was employed. Commissioned by the Royal Dutch Dental Association (KNMT), this agency, KBA in Nijmegen [27] takes care of the data collection and data processing for studies in dental practices. KBA is a member of a national Association for Policy Research [28] is ISO-certified (ISO-9001: 2008) and carries out the work activities in compliance with a legally mandatory data processing agreement.

## Results

After repeated requests, a total of 373 (40.4%) of the dentist registration forms and 372 (40.3%) of the patient questionnaires were returned. Data was available for 364 (39.4%) dentist-patient pairs. Table 1 describes the characteristics of older patients in this study.

**Table 1:** Characteristics of older people who visit the dental practice.

*demography*			*mean*	*sd*	*proportion*
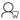			age		74.8	9.3	
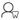	female*				52.8%
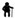	single*				33.9%
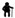	low socioeconomic status*				8.0%
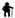	supplementary insurance*				70.8%
n = 359–373					
*morbidity and frailty*			*mean*	*sd*	*proportion*
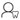	cardiovascular disease and respiratory disease*				47.1%
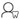	liver or gastrointestinal disease and kidney disease*				5.2%
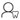	endocrine disease*				9.5%
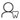	rheumatic disease*				9.5%
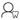	dementia and other neurological disease*				6.5%
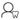	psychological disorder*				4.9%
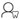	oncological disease*				4.6%
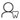	other disease*				12.5%
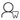	one or more diseases*				65.7%
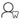	number of diseases			1.0	1.0
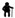	medication use*				75.2%
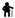	number of medications			2.9	3.1
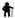	frailty*				9.6%
n = 353–367					
*lifestyle*			*mean*	*sd*	*proportion*
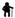	smoking*				9.4%
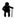	alcohol consumption*				78.2%
n = 360					
*oral care behaviour*			*mean*	*sd*	*proportion*
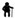	capable of oral self-care*				3.9%
n = 363					
*dental visits*			*mean*	*sd*	*proportion*
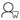	treatment intensity according to dentists (number of dental visits in past 2 years)		9.5	5.1	
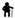	past 10 years regularly visited the dentist according to patient*				97.3%
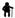	needed to visit the dentist according to patient*				97.3%
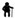	barrier experienced by patient to visit the dentist*				6.6%
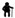	patient desires a better/ adjusted oral situation*				19.9%
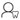	practice is wheelchair accessible according to dentist*				93.8%
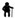	practice is accessible according to patient*				93.9%
n = 361–370					

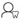
 registration form dentist.

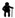
 patient questionnaire.

* dummy variable or dichotomous variable (yes/no); S2 Table provides an overview of the experienced barriers to visit the dentist of older people who visit the dental practice with respect to their oral situation and S3 Table an overview of the wishes of older people who visit the dental practice with respect to their oral situation.

The man/women ratio of dentists in the study was 63.0%/37.0%, and the average age was 49.7 years (SD = 10.8). Of these dentists, 45.3% live in the mainly urban western part of the Netherlands.

Table 2 illustrates that periodontal diseases (59.8%) and an estimated reduced ability to swallow (44.7%) were the most common oral problems observed by the dentists among older patients. Caries was observed in 21.3% of the patients. The older patients themselves most commonly mentioned pain (22.6%) and the aesthetics of their teeth (10.4%) as oral problems.

**Table 2:** Diagnosed oral problems by the dentist and experienced oral problems by older people, who visit the dental practice.

*diagnosed by dentist*	
periodontal problem	59.8%
estimated reduced ability to swallow	44.7%
wear	38.2%
caries (activity)	21.3%
loss of function	5.4%
residual roots	5.4%
aesthetic problem	3.8%
*experienced by patient*	
pain	22.6%
aesthetic problem	10.4%
chewing problem	3.5%
swallowing problem	1.6%
bad breath	0.0%
n = 353–373	

Table 3, which presents the overall assessment of oral health of older patients by dentists and older patients themselves is presented, illustrates that dentists assessed the oral health of older patients as good or acceptable in 51.4% of cases (expressed as the absence of diagnosed oral problems), whereas 76.2% of older patients considered their oral health to be ‘good’ or ‘acceptable’ (expressed as the absence of perceived oral problems).

**Table 3:** Level of oral health of older people who visit the dental practice, according to assessment dentist and assessment patient.

	*assessment dentist*		*assessment patient*	
good	*score 0*	12.8%	*score 0*	68.4%
acceptable	*scores 1–3*	38.6%	*scores 1–2*	7.6%
moderate	*scores 4–7*	40.9%	*scores 3–4*	21.8%
bad	*scores ≥8*	7.7%	*scores ≥5*	2.2%
mean	3.8		0.9	
mode	3.0		0.0	
median	3.0		0.0	
standard deviation	2.6		1.5	
minimum	0.0		0.0	
maximum	14.0		7.0	
n	337		360	
Spearman’s rho (ρ) with rating from dentist or patient	-0.411 (0.000)		-0.336 (0.000)	

From Table 4 it becomes clear that the dentist’s and the older patient’s assessments were comparable in 54.5% of cases, the older patient’s assessment was more positive in 35.0% of cases and the dentist’s assessment was more positive in 10.4% of cases.

**Table 4:** Level of oral health of older people who visit the dental practice, assessment dentist in relation to assessment patient.

*assessment dentist*	*assessment patient*	
	good/acceptable	moderate/bad
good/acceptable	40.9%^a^	10.4%^c^
moderate/bad	35.0%^b^	13.6%^a^
n = 337		

a. assessment dentist corresponds with assessment older patient.

b. assessment dentist indicates poorer oral health than assessment older patient.

c. assessment dentist indicates better oral health than assessment older patient.

Chi Square = 2.818, p = 0.093.

Table 5 shows that age, disease, medication use and frailty of older patients were related to a negative assessment of the oral health status by dentists. This also applies to difficulties with oral health self-care and the treatment intensity, expressed as the number of dental visits in the past two years. The assessment of the oral health status by the older patients themselves was related to gender, disease, medication use, frailty, having trouble with oral care, the desire to improve their oral status and the experienced accessibility of the dental practice.

**Table 5:** Correlation between characteristics of older people who visit the dental practice and the assessment of their oral health, according to respectively dentist and patient.

		*assessment oral health*					
		*according to dentist*			*according to patient*		
*demography*		*rho*	*p*	*n*	*rho*	*p*	*n*
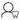	age	0.108	**0.024**	337	-0.033	0.267	360
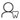	female*	-0.050	0.182	337	0.101	**0.028**	360
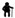	single*	-0.065	0.118	336	0.058	0.136	359
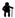	low socioeconomic status*	-0.048	0.191	337	0.007	0.445	358
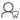	supplementary insurance*	0.006	0.458	334	0.030	0.289	356
*morbidity and frailty*		*rho*	*p*	*n*	*rho*	*p*	*n*
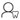	cardiovascular disease and respiratory disease*	0.226	**0.000**	331	0.020	0.355	354
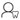	liver or gastrointestinal disease and kidney disease*	0.004	0.469	331	0.066	0.109	354
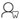	endocrine disease *	0.128	**0.010**	331	0.091	**0.044**	354
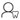	rheumatic disease *	0.091	**0.100**	331	0.032	0.272	354
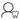	dementia and other neurological disease *	0.217	**0.000**	331	-0.007	0.447	354
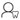	psychological disease *	0.169	**0.001**	331	0.148	**0.003**	354
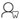	oncological disease *	0.027	0.311	331	0.023	0.335	354
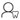	other disease*	0.063	0.125	331	0.024	0.328	354
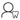	one or more disease *	0.305	**0.000**	331	0.057	0.144	354
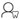	number of disease	0.331	**0.000**	331	0.092	**0.042**	354
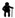	medication use*	0.226	**0.000**	337	0.090	**0.043**	363
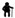	number of medications	0.316	**0.000**	337	0.109	**0.019**	363
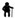	frailty*	0.107	**0.027**	328	0.088	**0.049**	357
*lifestyle*		*rho*	*p*	*n*	*rho*	*p*	*n*
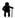	smoking*	0.053	0.116	336	-0.033	0.270	357
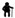	alcohol consumption*	-0.065	0.118	337	0.015	0.387	359
*oral care behaviour*		*rho*	*p*	*n*	*rho*	*p*	*n*
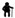	capable of oral self-care*	0.129	**0.009**	337	0.174	**0.000**	360
*dental visit*		*rho*	*p*	*n*	*rho*	*p*	*n*
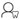	treatment intensity according to dentists (number of dental visits in past 2 years)	0.215	**0.000**	333	0.084	0.056	356
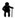	past 10 years regularly visited the dentist according to patient*	-0.181	**0.000**	337	-0.040	0.225	360
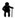	necessary to visit the dentist according to patient*	-0.063	0.123	337	-0.004	0.473	360
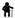	barrier experienced by patient to visit the dentist*	0.072	0.093	335	0.074	0.080	357
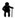	patient desires a better/ adjusted oral situation*	0.056	0.154	337	0.433	**0.000**	367
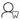	practice is wheelchair accessible according to dentist*	0.059	0.143	334	-0.028	0.299	357
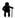	practice is accessible according to patient*	-0.003	0.477	336	-0.086	**0.053**	358

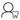
 registration form dentist.

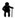
 patient questionnaire.

* dummy variable or dichotomous variable (yes/no).

The multivariate regression analysis shows that treatment intensity, number of dental visits, certain diseases and medication use determined the assessment of the oral health of older patients by the dentist (Table 6). That assessment became more negative in case of a higher treatment intensity and the more medication patients used, or if they had dementia and other neurological disorder and/or a cardiovascular or respiratory diseases. The assessment by the dentist was better for older people who had regularly visited the practice over the past ten years and had periodic oral examinations. Aforementioned variables explained over one fifth of the variance in the oral health assessed by the dentist.

**Table 6:** Multivariate correlation between characteristics of older people who visit the dental practice and the assessment of their oral health, according to dentist and patient.

assessment of oral health of older people, according to dentist						
		*estimate*	*std*. *error*	*Wald*	*df*	*p*
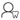	treatment intensity according to dentists (number of dental visits in past 2 years)	0.100	0.021	23.698	1	0.000
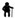	past 10 years regularly visited the dentist according to patient*	-2.725	0.694	15.430	1	0.000
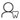	dementia and other neurological diseases*	1.545	0.415	13.838	1	0.000
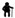	number of medications	0.129	0.037	11.863	1	0.001
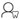	cardiovascular disease and respiratory disease*	0.477	0.228	4.375	1	0.036
*Nagelkerke Pseudo R-Square = 0*.*215*						
*Model Fitting Information*, *Chi-Square = 78*.*042*, *df = 5*, *p = 0*.*000/Goodness-of-Fit*, *Chi-Square = 2401*.*590*, *df = 2585*, *p = 0*.*995/Test of Parallel Lines*, *Chi-Square = 131*.*733*, *df = 65*, *p = 0*.*004*						
assessment of oral health of older people, according to patients themselves.						
		*estimate*	*std*. *error*	*Wald*	*df*	*p*
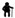	patient desires a better/ adjusted oral situation*	2.051	0.274	56.189	1	0.000
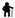	number of medications	0.105	0.035	8.753	1	0,003
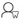	treatment intensity according to dentists (number of dental visits in past 2 years)	0.064	0.023	8.084	1	0.005
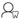	female*	0.512	0.252	4.127	1	0.042
*Nagelkerke Pseudo R-Square = 0*.*226*						
*Model Fitting Information*, *Chi-Square = 76*.*618*, *df = 4*, *p = 0*.*000/Goodness-of-Fit*, *Chi-Square = 1396*.*380*, *df = 1571*, *p = 0*.*999/Test of Parallel Lines*, *Chi-Square = 135*.*531*, *df = 24*, *p = 0*.*000*						

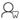
 registration form dentist.

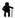
 patient questionnaire.

* dummy variable or dichotomous variable (yes/no), relative to no.

The older patients’ assessments of their oral health was also determined by treatment intensity and medication use: The higher the treatment intensity and the more medication they used, the less positively they assessed their oral health. Additionally, the assessment is poorer when older patients themselves desire a better oral situation. Compared to older males, older female patients estimated their oral health to be worse. These variables also explained over one-fifth of the variance in the oral health assessed by the older patients themselves.

## Discussion

This study showes that dentists assess the oral health of their older patients as ‘good’ or ‘acceptable’ in over half of the cases. The older patients themselves assessed their oral health as being in those two categories in over three- quarters of cases. In almost half of the dentist-patient pairings, the assessments of the dentists and patients did not match. Not only this study but also previous research has shown that there is a difference between objective treatment needs and subjective treatment needs in older people [29,30]. Such differences in assessment also occur in medical contexts. For instance, there was a difference between General Practitioners’ (GP’) assessment of perceived frailty and the self-reported frailty by older people [31]. Studies on self-reporting of chronic diseases in older people versus data from GPs showed older people both over-reporting and under-reporting diseases. Underlying factors were gender, age and SES as well as the mental status of the older person. Poor communication between physicians and other care providers and patients also played a role [32–34].

In this study, treatment intensity and medication use were related to the assessment of the oral health for both dentists and older patients. The higher the treatment intensity and the more medication older patients used, the more negative the assessment of their oral health was by both the dentists and the older patients themselves. When older patients stated they had visited the dentist regularly in the past ten years for periodic oral examinations, the dentist assessed better oral health. For the dentist, the relation was also determined by diseases l such as dementia and other neurological conditions, cardiovascular diseases and respiratory diseases, while for the older patients, gender and their desired oral status appeared to be partly decisive for the assessment of their oral health.

Dental visits help maintain good oral health. Research has shown that the frequency of dental visits decreases with age [35] although it is important that older people keep visiting the dentist regularly, in particular when they become less healthy and may possibly be more dependent on care. This is a particular concern for frail older people because there is often a ‘geriatric triad’, in that somatic, psychiatric and social problems interwaeve. This interwovenness makes it difficult to establish a direct relationship between the main complaint and the symptoms, as is the case with a single disorder [36]. It is, therefore, important to view the oral health of the older person as a part of and in relation to general health [37,38].

In addition, the higher the treatment intensity was, the poorer the assessment of the old patient’s oral health by the dentist. For those patients, treatment was problaby necessary for maintaining comfort and avoiding pain. In any case research shows that older people mainly visit the dentist for restorative procedures, followed by prosthetics and periodontal treatment [39–41]. These findings suggest that the mouths of older people retain traces of previous oral health problems that may require more restorative work and functional repair. This would argue in favour of deploying early prevention from a younger age and encouraging people to maintain oral health when getting older. This means taking oral health into account at every stage of life and considering it in relation to the general health of an older person [42–44].

Another deciding factor for the dentist in assessing of the oral health of older people is whether the older people have been ill or not, expressed as the number of diseases and the use of medication. The literature shows relationships between oral health and a numerous disorders [38]. Strong associations are observed between infections such as periodontitis and cardiovascular disorders, but this cannot be seen as a causal relationship [45]. In dementia, it has been hypothesised after research in mice that the bacterium *Porphyromonas gingivalis* could play a role in the development of Alzheimer’s disease [46]. More research into the possible relationship between oral health and cognitive decline is needed and could help raise awareness of the importance of preserving a good oral health status [47,48] It has also been found that oral problems are seen in older people who use medication [49], but this study revealed it as a factor, that appeared to be strongly related to the poor oral health of older people. Caregivers in general, and dentists in particular, should be alert to the risks of specific hyposialia-inducing medications and polypharmacy on oral health.

In older patients, gender and the desire to improve oral status also determined the assessment of oral health. Regarding gender, a study into dental visits in the Netherlands shows that women visit the dentist more often than men [35,50]. Other research showed that men, in general, cared less about natural teeth than women [51]. Furthermore, Hakeberg and Wide Boman (2017) showed in their study on self-reported oral health and general health in relation to socioeconomic position that women rate their (oral) health significantly more poorly than men [52,53]. Regarding the relation with wishes for their oral health, it is conceivable that a wish to change their oral situation is prompted by a less experienced oral health status.

This study has related strengths and limitations. To our knowledge, this is the first time that the investigation of oral health was approached broadly and holistically. This approach, based on the FDI definition of oral health, is well suited to the older patient. For them, particularly in cases of frailty, overall health, psychological and social problems are often interwoven as well. However, the FDI definition of oral health given by the FDI was not used to create the questionnaire. The study began at the end of 2016 or early 2017, whereas the definition by the FDI was published in the second half of 2016 after the questionnaire had already been drawn up. Certain aspects of oral health may not have been adequately covered because they were not explicitly asked about. This means that the measures constructed for oral health in this study could not be externally validated. However, these measures appeared to be internally valid, according to the relation with the oral health scores for older patients that the dentists and the patients themselves gave. The measures, as constructed here, reflect upon the oral health of older people who still visit the dental practice rather than upon the older people in the Netherlands as a whole. The fact that older people with a low SES are underrepresented in this study is an indication of this. It is known that in the Netherlands this group of older people often do not any longer visit the dentist [30].

Overall, this study finds that the oral health of older people involves more than the absence of dental problems. A better view was obtained on the oral health of older people in the dental practice and it was possible to demonstrate some aspects and circumstances that play a decisive role in this, from the distinct outlook of the dentists and the older patients themselves.

For the general practitioner, this outcome offers the following points of interest when providing oral health care to older patients:

- Older patients tend to view their oral health status more positively than dentists do. Older female patients may express more concern regarding their oral health status than older male patients.
- Chronically illness as expressed by the number of diseases and the use of medication, seems to be a risk factor for poor oral health.
- It is likely that community dwelling older people who no longer visit the dentist due to morbidity, limitations, SES and disabilities are at serious risk for oral health problems.
- Regular dental visits or (if needed) home visits by the oral healthcare provider seem decisive for maintaining oral health.

## Supporting information

S1 Table Ranking of oral problems according to dentist and older patient in the interest of the oral health of older people who visit the dental practice. (DOCX)

S2 Table Experienced barriers to visit the dentist of older people who visit the dental practice with respect to their oral situation. #1 multiple barriers possible.
(DOCX)

S3 Table Wishes of older people who visit the dental practice with respect to their oral situation. #1multiple improvement or adjustment possible.
(DOCX)
