# Supplementary material for: Assessed and perceived oral health of older people who visit the dental practice, an exploratory cross-sectional study
Source: PLoS One. 2021 Sep 24;16(9):e0257561. doi: 10.1371/journal.pone.0257561 (PMC8462729; doi:10.1371/journal.pone.0257561)
Supplement: S1 Table — (DOCX) [file pone.0257561.s001.docx]

**S1 Table. Ranking of oral problems according to dentist and older patient in the interest of the oral health of older people who visit the dental practice.**

| **S1** |  | | | | |
| --- | --- | --- | --- | --- | --- |
| *according to dentist (n=23)* | | average rank number | sd | min/max | weighting factor |
| caries (activity) | | 2.70 | 1.3 | 1/6 | 3 |
| loss of function | | 2.78 | 1.9 | 1/6 | 3 |
| periodontal problem | | 3.39 | 1.9 | 1/7 | 3 |
| estimated reduced ability to swallow | | 4.87 | 2.0 | 1/8 | 2 |
| residual roots | | 5.00 | 2.1 | 1/8 | 2 |
| aesthetic problem | | 6.04 | 2.0 | 1/8 | 1 |
| wear | | 7.26 | 1.0 | 1/8 | 1 |
| *according to patient (n=10)* | |  |  |  |  |
| pain | | 2.4 | 1.5 | 1/5 | 3 |
| chewing problem | | 2.4 | 1.0 | 1/4 | 3 |
| swallowing problem | | 2.9 | 1.4 | 1/5 | 2 |
| bad breath | | 3.2 | 1.6 | 1/5 | 2 |
| aesthetic problem | | 4.1 | 1.1 | 2/5 | 1 |
