# Supplementary material for: Assessed and perceived oral health of older people who visit the dental practice, an exploratory cross-sectional study
Source: PLoS One. 2021 Sep 24;16(9):e0257561. doi: 10.1371/journal.pone.0257561 (PMC8462729; doi:10.1371/journal.pone.0257561)
Supplement: S2 Table — #1 multiple barriers possible. (DOCX) [file pone.0257561.s002.docx]

**S2 Table. Experienced barriers to visit the dentist of older people who visit the dental practice with respect to their oral situation.** #1 multiple barriers possible

| **S2** |  | | | | |
| --- | --- | --- | --- | --- | --- |
| no barriers | |  |  | 345 | 93.5% |
| barriers^#1^ | |  |  | 24 | 6.5% |
| - takes a lot of effort | | *2* | *0.5%* |  |  |
| - problems with guidance | | *3* | *0.8%* |  |  |
| - problems with transportation | | *14* | *3.8%* |  |  |
| - problems with health | | *5* | *1.3%* |  |  |
| - fear | | *6* | *1.6%* |  |  |
| n = 372 | | | | | |
|  | | | | | |
