# Supplementary material for: Assessed and perceived oral health of older people who visit the dental practice, an exploratory cross-sectional study
Source: PLoS One. 2021 Sep 24;16(9):e0257561. doi: 10.1371/journal.pone.0257561 (PMC8462729; doi:10.1371/journal.pone.0257561)
Supplement: S3 Table — #1multiple improvement or adjustment possible. (DOCX) [file pone.0257561.s003.docx]

**S3 Table. Wishes of older people who visit the dental practice with respect to their oral situation.**

#1 multiple improvement or adjustment possible

| **S3** |  | | | | |
| --- | --- | --- | --- | --- | --- |
| no wishes | |  |  | 109 | 29.3% |
| maintain oral situation | |  |  | 189 | 50.8% |
| improvement and/or adjustment^#1^ | |  |  | 74 | 19.9% |
| - improve chewing function | | *9* | *2.4%* |  |  |
| - aesthetic wishes | | *36* | *9.7%* |  |  |
| - improve prosthesis fit | | *21* | *5.6%* |  |  |
| - slowly working towards prosthesis | | *15* | *4.0%* |  |  |
| n = 372 | | | | | |
|  | | | | | |
